# THE ASSOCIATION BETWEEN PERCEIVED SOCIAL SUPPORT AND LIFE SATISFACTION: RACE/ETHNICITY DISPARITIES

**DOI:** 10.1093/geroni/igae098.2432

**Published:** 2024-12-31

**Authors:** Kingsley Mbam, Yan-Jhu Su, Wuyi Dong

**Affiliations:** University of Massachusetts Boston, Boston, Massachusetts, United States; University of Massachusetts Boston, Boston, Massachusetts, United States; University of Massachusetts Boston, Boston, Massachusetts, United States

## Abstract

**Objective:**

Studies have shown an association between perceived social support and life satisfaction. However, fewer studies covered the racial/ethnic disparities in this association. This study looks to address this gap by assessing how perceived social support differs across racial and ethnic groups and their life satisfaction.

**Methods:**

This cross-sectional study used data from the 2020 Health and Retirement Study (HRS). The respondents include individuals 55 and older without any missing survey responses (n=1,901). The perceived social support was assessed using the summary score of social integration, which included spousal/partner support, children support, friend support, and non-nuclear family support. Life satisfaction was assessed using Diener’s measure of life satisfaction. Control variables, including demographic characteristics, household income, and self-rated health, were added to this analysis. Ordinary least squared (OLS) regression was implemented to examine whether race/ethnicity moderates the association between perceived social support and life satisfaction.

**Results:**

Adjusted models show results disclosing that people with more perceived social support had significantly higher life satisfaction (b = 0.32, p < 0.001), and interaction effects showed that the association among Hispanic people is significantly stronger compared to non-Hispanic Whites (b = 0.17, p = 0.03).

**Discussion:**

These results suggest that improving social support for individuals age 55 and older across racial groups can enhance their life satisfaction, which is essential to improving their overall health and well-being. Furthermore, this study widens the understanding of the demographic factors that influence the association between perceived social support and life satisfaction.

